# A63 TRENDS AND OUTCOMES OF LIVER DISEASE HOSPITALIZATIONS DURING THE CORONAVIRUS PANDEMIC IN THE UNITED STATES: A NATIONWIDE POPULATION-LEVEL ANALYSIS

**DOI:** 10.1093/jcag/gwac036.063

**Published:** 2023-03-07

**Authors:** M Sedarous, M Youssef, A D Adekunle, O Babajide, M Rubens, P N Okafor

**Affiliations:** 1 Division of Gastroenterology, Queen's University, Kingston; 2 Division of Internal Medicine, University of Toronto, Toronto, Canada; 3 Division of Internal Medicine, St. Luke’s Hospital, Chesterfield; 4 Division of Internal Medicine, One Brooklyn Health, Brooklyn; 5 Office of Clinical Research, Miami Cancer Institute, Miami; 6 Division of Gastroenterology and Hepatology, Mayo Clinic, Florida, United States

## Abstract

**Background:**

The impact of the Coronavirus disease-2019 (COVID-19) pandemic on patients with liver disease is not well described at the population level in the United States.

**Purpose:**

We used the largest, nationwide inpatient dataset to describe inpatient liver disease outcomes in the United States during the first year of the pandemic (2020) using 2018 and 2019 as comparator years.

**Method:**

Using the National Inpatient Sample (2018-2020), we explored year-to-year and 2020 month-to-month trends in hospitalizations, length of stay, and inpatient mortality for liver-related indications including compensated cirrhosis, decompensated cirrhosis, alcohol-associated liver disease (ALD), alcohol-associated hepatitis (AH), hepatocellular carcinoma (HCC), and variceal upper gastrointestinal bleeding (VUGIB) using regression modeling. We also looked at the impact of the COVID-19 pandemic on liver transplantation rates. A p-value <0.05 was considered statistically significant.

**Result(s):**

Hospitalizations for both compensated and decompensated cirrhosis decreased in 2020 compared to 2019 (relative change [RC] of 1.5%, p <0.001, Table 1). Interestingly, hospitalizations for ALD and AH increased in 2020 compared to pre-pandemic years (ALD RC=15.5% and AH RC 17.0%; p<0.001). Despite the decrease in cirrhosis hospitalizations in 2020, all-cause inpatient mortality among patients with compensated cirrhosis increased from 30,135 in 2019 to 35,220 in 2020 (p<0.001) and from 22,850 in 2019 to 26,390 in 2020 among patients with decompensated cirrhosis (p<0.001). This was accompanied by a 27.8% increase in mortality for ALD (p=0.004) in comparison to pre-pandemic years. Corresponding to the peaks of the pandemic, we observed the fewest cirrhosis hospitalizations in April and December 2020 (Table 2), however, these months had the highest observed mortality rates (p-trend ≤ 0.004). Reassuringly, liver transplantation rates were not significantly impacted by the COVID-19 pandemic (p=0.51).

**Image:**

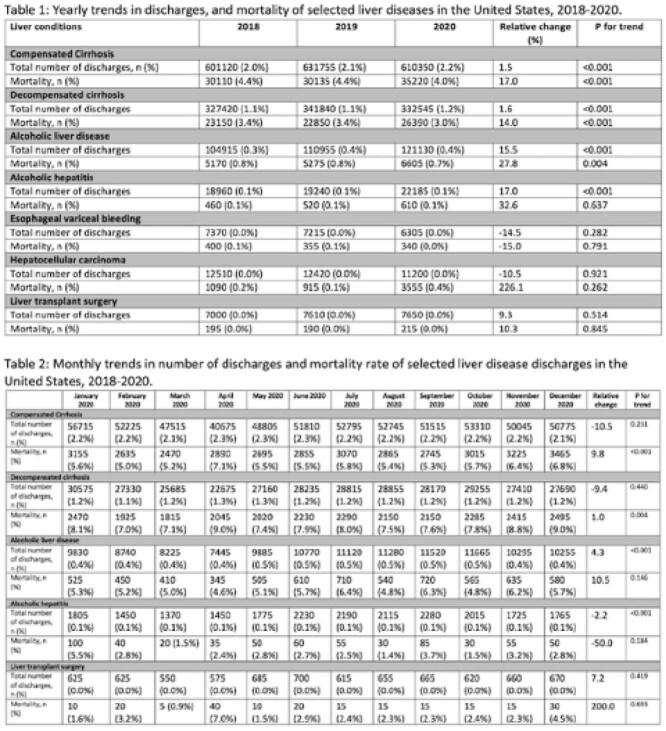

**Conclusion(s):**

Cirrhosis hospitalizations, in general, decreased in 2020 compared to pre-pandemic years but were associated with higher all-cause mortality rates particularly in the peak months of the COVID-19 pandemic (April and December 2020) possibly reflecting COVID-19 specific mortality. Alcoholic liver disease admissions also increased during the pandemic while liver transplantation rates were not significant impacted.

**Please acknowledge all funding agencies by checking the applicable boxes below:**

None

**Disclosure of Interest:**

None Declared

